# Quitting Your Day Job in Response to Stress: Cell Survival and Cell Death Require Secondary Cytoplasmic Roles of Cyclin C and Med13

**DOI:** 10.3390/cells14090636

**Published:** 2025-04-25

**Authors:** Justin R. Bauer, Tamaraty L. Robinson, Randy Strich, Katrina F. Cooper

**Affiliations:** Department of Cell and Molecular Biology, School of Osteopathic Medicine, Rowan-Virtua College of Medicine and Life Sciences, Rowan University, Stratford, NJ 08084, USA; bauerj48@rowan.edu (J.R.B.); robins196@rowan.edu (T.L.R.); strichra@rowan.edu (R.S.)

**Keywords:** Mediator kinase module, cyclin C, Med13, mitochondrial hyperfission, cell death, P-body, cargo hitchhiking autophagy

## Abstract

Following unfavorable environmental cues, cells reprogram pathways that govern transcription, translation, and protein degradation systems. This reprogramming is essential to restore homeostasis or commit to cell death. This review focuses on the secondary roles of two nuclear transcriptional regulators, cyclin C and Med13, which play key roles in this decision process. Both proteins are members of the Mediator kinase module (MKM) of the Mediator complex, which, under normal physiological conditions, positively and negatively regulates a subset of stress response genes. However, cyclin C and Med13 translocate to the cytoplasm following cell death or cell survival cues, interacting with a host of cell death and cell survival proteins, respectively. In the cytoplasm, cyclin C is required for stress-induced mitochondrial hyperfission and promotes regulated cell death pathways. Cytoplasmic Med13 stimulates the stress-induced assembly of processing bodies (P-bodies) and is required for the autophagic degradation of a subset of P-body assembly factors by cargo hitchhiking autophagy. This review focuses on these secondary, a.k.a. “night jobs” of cyclin C and Med13, outlining the importance of these secondary functions in maintaining cellular homeostasis following stress.

## 1. Introduction

Under normal physiological conditions, cells attain homeostasis through complex regulatory events, including balancing the transcription and translation of proteins with their degradation. Transcriptional control is mediated through the activities of DNA binding transcription factors, RNA polymerase II (RNAPII), RNAPII associating factors, and chromatin remodeling enzymes. Protein degradation is mediated by the ubiquitin proteasome system (UPS) and many macroautophagy (hereafter autophagy) pathways. Understanding the molecular details of how cells balance these events is crucial, as disrupting these pathways results in a homeostatic disruption. Moreover, as the efficiency of degradation pathways deteriorates with age, such imbalances are linked with age-related pathologies, including cancer and proteinopathies [1,2,3,4,5,6,7]. For example, dysfunctional degradative pathways allow misfolded proteins to accumulate, eventually forming aggregates that are characteristic of neurodegenerative diseases, including Alzheimer’s and amyloid lateral sclerosis (ALS) [8,9].

Cells constantly encounter environmental or physiological stress, including osmotic, oxidative, thermal, and nutritional stressors [10]. After exposure, cells face a critical decision: do they adapt and survive or initiate regulated cell death pathways such as necrosis or apoptosis? Adaptation involves reprogramming transcription, translation, and protein degradation pathways. Due to the high degree of conservation among these processes, the budding yeast *Saccharomyces cerevisiae* has been an excellent model for understanding the molecular stress response. Studies on this single-celled eukaryote have provided valuable insights into many aspects of mammalian biology. For instance, autophagy was first identified in yeast and later found to be highly conserved across species [11]. Furthermore, yeast has been instrumental in characterizing genetic pathways linked to neurodegenerative diseases such as ALS, Parkinson’s, or Alzheimer’s [12,13,14,15,16,17].

Regardless of the chosen survival or death pathway, cells must coordinate various activities to achieve the desired outcome. For example, a survival response upregulates chaperones to help protein refolding, induces autophagy to recycle macromolecules, and increases mitochondrial fusion to enhance ATP production. Conversely, the cell death response stimulates pro-death gene transcription, induces mitochondrial fission, and coopts autophagy genes for cell death functions [18]. However, the connection between survival and cell death pathways is complicated with shared regulators and seemingly shared responses [18].

**Figure 1:**
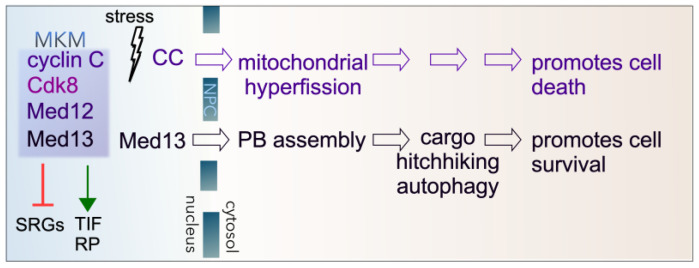
Cytoplasmic roles of cyclin C (CC) and Med13 following stress in yeast. The Mediator kinase module (MKM) predominantly represses stress response genes (SRGs) under normal physiological conditions. It also positively regulates a subset of genes encoding translation initiation factors (TIFs) and ribosomal proteins (RPs). Following ROS stress, cyclin C translocates to the cytoplasm, which is required for mitochondrial hyperfission. Before destruction by the UPS, cyclin C also promotes regulated cell death by unknown mechanisms. Following starvation stress, Med13 translocates to the cytoplasm, playing a role in P-body (PB) assembly. It is also required for the autophagic degradation of a subset of P-body assembly factors by cargo hitchhiking autophagy. The translocation of Med13 following starvation stress is necessary for cell survival.

This review focuses on the activities of the Mediator kinase module (MKM), previously called the Cdk8 kinase module, a complex whose components directly coordinate gene transcription with cytoplasmic events to coordinate survival and death cell fate decisions (Section 2 and Section 3). In short, cyclin C and Med13 (two members of the MKM) have secondary cytoplasmic roles following cell death and cell survival cues, respectively (Figure 1). In the cytoplasm, they interact with a host of cell death and cell survival proteins, including the newly defined cargo hitchhiking autophagy pathways (Section 4, Section 5, Section 6 and Section 7) [19]. This review outlines the importance of these secondary functions of cyclin C and Med13 in maintaining cellular homeostasis following stress and diseases associated with misregulation (Section 5). Throughout, yeast proteins are written with a capital followed by lowercase letters (e.g., Cdk8), whereas all capitals are used to describe mammalian proteins (e.g., CDK8).

## 2. The Mediator Kinase Module

### 2.1. Structure and Function of the MKM

#### 2.1.1. MKM Structure

**Figure 2:**
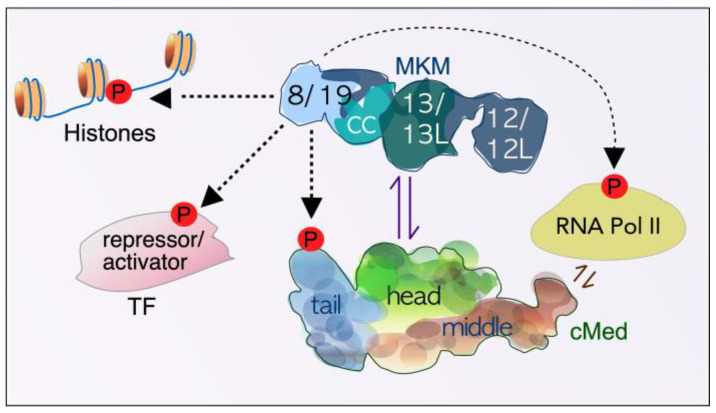
Schematic overview depicting the relationship between the dissociable Mediator kinase module (MKM), the core Mediator (cMed), and other transcription-associated factors. Under normal physiological conditions, the MKM alone, or attached to cMed, phosphorylates many targets to positively and negatively regulate transcription. Paralogs exist for all subunit components except for cyclin C (CC). 8—CDK8, 19—CDK19, 12—Med12, 12L—Med12L, 13—Med13, 13L—Med13L TF—transcription factor, P—phosphorylation event.

The Mediator comprises a large core 26-subunit Mediator complex (cMED) and a dissociable Mediator kinase module (MKM), which functions as a critical coregulator of RNA polymerase II (RNAPII) transcription (Figure 2) [20,21,22,23,24,25]. As such, the Mediator complex exists as two distinct entities, depending on whether it is bound to the MKM. The MKM is a highly conserved complex consisting of four proteins: cyclin C, its cognate kinase CDK8, and two structural proteins, MED12 and MED13, in a 1:1:1:1 stoichiometric ratio [26]. In mammals, the MKM contains paralogues of its members, except for cyclin C, namely MED12L, MED13L, and CDK19 [27]. Unlike the founding members of the cyclin-dependent kinase family, cyclin C–CDK8 does not mediate cell cycle progression [22,28].

CDKs are typically activated by cyclin association and phosphorylation in the T-loop domain by the CDK-activating kinase (CAK) [29]. However, CDK8 and CDK19 lack this canonical phosphorylation residue suggesting a different mechanism is in place. Previous studies found a role for MED12 in stabilizing the CDK8 or CDK19 T-loop in an active conformation [26,30]. In addition, a recent cryo-electron microscopy structure of the yeast MKM also revealed that Med13’s bilobal architecture resembles that of argonaut proteins [31]. While this would imply a new role for Med13 in nucleic acid binding, the lack of several critical conserved catalytic residues found in other argonaut proteins adds complexity to Med13’s role in transcription and mRNA metabolism.

#### 2.1.2. Function of the MKM in Yeast

The initial identification of the MKM components in yeast was conducted through genetic screens, which searched for mutations that allowed aberrant upregulation of a variety of genes. This formally indicated a role for the MKM in gene repression. Subsequent molecular analyses confirmed this model with the MKM repressing many genes during normal growth that are induced in response to oxidative stress, sucrose utilization, autophagy, and meiotic development [21,32,33,34,35,36,37,38,39,40,41,42,43,44]. Genomic studies confirmed that the MKM played a predominantly negative role in transcription [36,39]. Precise mechanistic details of how the MKM-Mediator regulates gene expression are still emerging [45]. Cdk8 represses transcription through phosphorylating and subsequently inhibiting transactivators. For example, Cdk8 phosphorylation prevents nuclear accumulation of Msn2 and destabilizes Ste12 and Gcn4 [46,47]. In addition, the MKM inhibits pseudohyphal growth by preventing H3K4 trimethylation at the *FLO11* promoter [48]. Finally, Cdk8 modification of the great wall protein kinase Rim15 increases its nuclear export [43]. These studies highlight an overall strategy for MKM-dependent phosphorylation stimulating relocalization or degradation of transcription factors to enforce repression. This theme is repeated in mammalian cells.

Although it is a relatively minor role, the MKM is also involved in transcriptional activation. Early studies revealed a positive role for the yeast MKM in phosphorylating TFs responsible for changes in carbon and nitrogen utilization [42,47]. Similar to its repression function, the activated transcription factors, Gal4 and Ste12, are required when cells shift to an alternative carbon source coupled with reduced nitrogen. However, we recently discovered that the MKM also positively regulates a subset of genes encoding ribosomal proteins (RPs) and translation initiation factors (TIFs) under normal physiological conditions [49]. This mechanism demonstrates the conserved nature of MKM activity, as cyclin C is necessary for maintaining steady-state levels of a subset of ribosomal genes in mouse and human cell lines [49]. The repression of this subset of translation-associated genes occurs in response to stress and is dependent on MKM disassembly [49]. This process makes sense as ribosome biogenesis consumes over 60% of cellular energy and resources [50]. Reducing ribosome biosynthesis helps conserve energy to enhance cellular survival during stress [51].

#### 2.1.3. Function of the MKM in Mammalian Cells

Similar to yeast, the MKM both positively and negatively regulates genes involved in the stress response, differentiation, and metabolism [42,52,53,54,55,56,57,58]. However, in mammalian cells, the split between repressed and activated genes is approximately 50:50 [52]. Several models have been put forward to explain the repressor function of the mammalian MKM. For example, biochemical reconstitution experiments and molecular modeling have suggested that the MKM sterically inhibits the Mediator from interacting with RNAPII at promoters [20,22,26,59,60,61,62,63,64,65]. Other methods exist for MKM-dependent repression of gene transcription. For example, CDK8 phosphorylates cyclin H, inhibiting TFIIH activity [66]. In addition, CDK8 phosphorylation induces destruction of the Notch receptor intracellular domain (ICD), inhibiting activation of this pathway [67]. Similar to yeast studies, the mammalian MKM has also been implicated in repression through regulating chromatin modifications. For example, CDK8 is required for Xist-dependent X chromosome silencing in mice [68]. Finally, unbound MKM has been reported to regulate transcription independently of the cMed [26,69]. However, it remains unclear if this function is mediated by the MKM–Mediator complex or the MKM acting as an independent entity [70,71,72].

The role of the MKM in directing transcription in response to external cues has been the subject of several recent reviews [65,72,73] and therefore will not be discussed in detail. Briefly, cyclin C and its cognate kinase are constitutively active in normal unstressed conditions, where they phosphorylate several factors to both positively and negatively regulate transcription [42,53,54,70,71,72,74,75,76,77,78,79,80,81,82]. Key among these targets are many transcription factors (TFs), including STAT-1 and SREBP-1 [53,54,55,56,57]. In addition, the MKM kinase phosphorylates histone H3 at serine 10 (H3S10) [26]. This modification is associated with transcriptional activity [83] as it prevents a repressive methylation mark [84].

In mammals, the MKM controls transcription through both the proximal promoter region and by modifying proteins at super-enhancer sites. Super-enhancers are regions further upstream of the proximal promoter, containing an array of TF binding sites [72,85]. Although the exact contribution of each of these individual sites to gene expression is not clear, it is proposed that they directly interact with RNAPII holoenzyme components through the looping of the chromatin. The specific role of the MKM at the super-enhancers again focuses on modifying TF and/or chromatin [73]. For example, inhibiting CDK8 and CDK19 results in both upregulation and downregulation in super-enhancer-controlled genes in acute myeloid leukemia cells [86]. In addition, chromatin immunoprecipitation (ChIP) studies confirmed that the MKM was resident at these control regions. Formally, these results argue that the MKM either represses or stimulates transcription, depending on the locus. In this scenario, MKM regulatory specificity would be derived from TFs or other chromatin modifying enzymes present. A recent study found that genes induced by the interferon response in cells derived from Down’s syndrome patients are suppressed by treatment with a potent CDK8/CDK19 inhibitor [87]. Although not mutually exclusive, another potential role for the MKM is employing the IDR domains of MED12 and MED13 to establish a liquid–liquid phase separation (LLPS) or biomolecular condensate that increases substrate and enzyme concentrations around promoters [88]. A role for MKM components in establishing such a specialized environment has already been observed in the cytoplasm (Section 3 and Section 4). In summary, the MKM influences important cell fate decisions through modifying a myriad of transcription-related targets that direct cellular and developmental homeostasis.

### 2.2. Regulation of the MKM

#### 2.2.1. Dynamic MKM Promoter Recruitment and Expulsion

Structural and biochemical studies found that the MKM–Mediator association is a dynamic, reversible process. The MKM concentration is reported to be 10-fold lower than the cMed, consistent with the finding that the Mediator is often found at promoters without the MKM. This dynamic aspect of the MKM was underscored using ChIP experiments to examine promoter occupancy before and after oxidative stress. In these studies, when MKM repressor function needs to be relieved, it is removed from the promoter. Conversely, the MKM is recruited to promoters in which it plays a transcriptional activator role following stress [52]. However, a small subset of promoters show disparity in MKM component association, with cyclin C being absent while CDK8, MED13, and MED13L remained. This may provide a mechanism that allows ~20% of cyclin C to leave the nucleus in response to stress.

In yeast, the situation is different. Rather than the MKM moving as a unit to different promoters, this complex is completely disassembled in response to oxidative or nutritional stress. As detailed below, this disruption allows MKM components to perform their “night jobs” in the cytoplasm. Currently, the underlying mechanism directing recruitment or expulsion of the MKM from promoters is only partially understood (see [73] for a recent review). Although it remains unclear how the MKM–Mediator interaction is controlled in yeast, it has been suggested that Cdk8 phosphorylation of cMed components reduces MKM binding [60,73,89]. Given the seemingly constitutive nature of Cdk8 activation, this suggests that either the MKM is always unstably associated with the cMed or there are other mechanisms for maintaining this interaction when necessary. Finally, the stability of the MKM components is also the subject of regulation. For example, MED13 and MED13L are targeted for degradation by the SCF-Fbs7 E3 ubiquitin ligase in mammals [62]. In addition, loss of Cdk8 results in destabilization of cyclin C in yeast [81,90].

#### 2.2.2. MKM Disassembly Following Cell Death Cues Triggered by ROS

In response to oxidative stress, cyclin C, but not Cdk8, translocates to the cytoplasm, resulting from MKM disassembly [91]. This event is conserved in mammals [92,93]. In yeast, the molecular details of MKM disassembly in response to ROS involve an intertwined network of phosphorylation and ubiquitination events. The stress signal is transmitted by the conserved MAPK of the Cell Wall Integrity (CWI) pathway to Slt2 (Mpk1, (ERK5 ortholog, [94,95]) that directly phosphorylates cyclin C and Med13 [96,97,98,99]. Intriguingly, Kdx1 (Mlp1), the pseudokinase of the CWI pathway, is also required for cyclin C release [98]. Kdx1 interacts with the transcription factor Ask10 and likely induces cyclin C translocation to the cytoplasm indirectly [97,99]. CWI-triggered phosphorylation of Med13 results in its destruction by UPS mediated by the E3 ligase complex SCF^Grr1^ [99,100]. In addition, cyclin C is directly phosphorylated at a single site (S266). The net outcome is MKM disassembly and cyclin C nuclear release [91,101]. In yeast, the UPS destroys cytoplasmic cyclin C following its role in mitochondrial hyperfission [33,34,101].

Genetic studies showed that Med13 destruction is required for cyclin C nuclear release. In addition, Med13 destruction requires a priming event mediated by Cdk8 in unstressed cells [99]. Thus, recognition of the Med13 degron uses two phosphorylation marks, one to prime the degron, the second for its recognition by ubiquitin ligases [102,103]. This ensures that the nuclear release of cyclin C is the correct response to the environmental input. Additionally, direct phosphorylation of Med13 by Snf1, a highly conserved adenosine monophosphate-activated protein kinase (AMPK) that is activated in response to a variety of stresses, is required for cyclin C’s translocation to the cytoplasm [104,105].

Taken together, the studies discussed above suggest that Med13 acts as a physical tether, keeping cyclin C in the nucleus. Consistent with this, in the absence of Med13, cyclin C is aberrantly released into the cytoplasm, where it induces mitochondrial fission in the absence of stress [100]. Furthermore, deletion of the holoenzyme associating domain (HAD), the domain on cyclin C that interacts with Med13 [106], also results in the precocious release of cyclin C from the MKM. Loss of Med13 interaction leads to constitutive cytoplasmic localization of cyclin C and mitochondrial fission in the absence of stress [100]. The HAD is highly conserved, being required for the cyclin C–Med13 interaction in mammalian cells [107]. Based upon these studies, we designed a peptide mimetic of this domain (S-HAD) which stimulates cyclin C nuclear release in mammalian cells in the absence of stress, highlighting the conservation of both the domain and function [107].

#### 2.2.3. MKM Disassembly Following Cell Survival Cues Triggered by Nitrogen Starvation

In the yeast model system, nitrogen starvation activates both bulk and cargo hitchhiking autophagy pathways. Bulk autophagy is essential for recycling cellular components, producing energy, and ensuring survival [108]. In contrast, cargo hitchhiking autophagy is a selective pathway that degrades specific substrates, including Med13, certain ribosomal proteins, and components involved in P-body assembly [19]. Given that the MKM represses many crucial autophagy genes, it is not surprising that effective induction of autophagy requires its disassembly [44,109]. Following nitrogen starvation cyclin C does not translocate to the cytoplasm. Instead, the ubiquitin–proteasome system (UPS) targets and degrades cyclin C in the nucleus via an unidentified E3 ligase [19,44]. This response is vital for survival, as it prevents cyclin C from inducing mitochondrial hyperfission and regulated cell death [109]. Thus, the mitochondria remain hyperfused, a morphology associated with maximal ATP production [4,110]. MKM disassembly following nitrogen starvation also results in Med13 translocating to the cytoplasm [44,111]. Here, it is required for P-body assembly, and the autophagic degradation of a subset of P-body assembly factors by cargo hitchhiking autophagy [19,112].

How the stress signal is transmitted to the MKM following nitrogen starvation remains unknown. However, it is known that in yeast that the activity of Slt2 is activated in response to the Target of Rapamycin Complex 1 (TORC1) inhibition [113]. However, phosphorylation of cyclin C by Slt2 is not required for its degradation following nitrogen starvation [109]. This suggests that a different protein, possibly Med13, is the target of Slt2 activity following starvation stress. These studies highlight how the MKM responds to cell death and cell survival cues, even though the CWI is activated in both cases. Interpretation of the input signal is critical as expressing a fusion protein in which cyclin C is tethered to the outer mitochondrial membrane results in mitochondrial fission and the execution of cell death pathways following cell survival cues [109].

## 3. Roles of Cyclin C in the Cytoplasm

### 3.1. The Role of Cyclin C Stress-Induced Mitochondrial Hyperfission

It is well documented that mitochondria are dynamic organelles with distinct morphologies within and among cell types and tissues (see [114] for a recent comprehensive review). Furthermore, mitochondrial function and morphology are intricately linked, with dynamics playing a role in many processes, including ATP production, calcium homeostasis, and RCD [115]. The remodeling of mitochondria morphology occurs through the regulation of two opposing processes, mitochondrial fission and fusion, both of which are highly dependent on cell type and the functional state of mitochondria [116,117].

#### 3.1.1. Two Classes of Mitochondrial Fission

**Figure 3:**
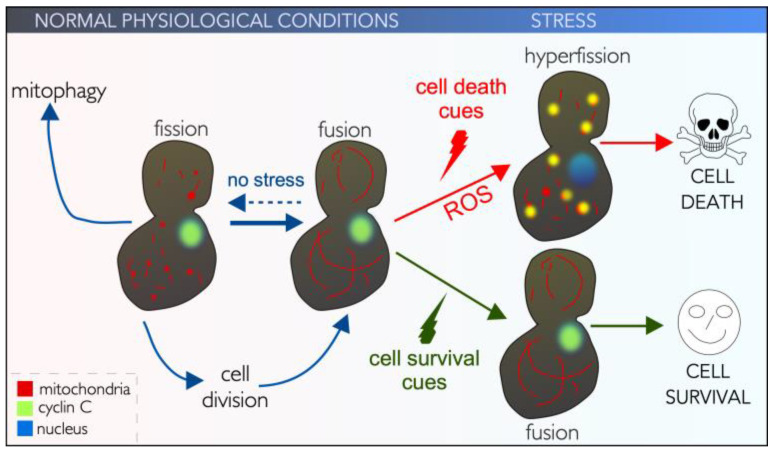
Mitochondrial morphology is linked to cell survival and cell death. See text for details.

From yeast to metazoans, mitochondrial fusion and fission often occur simultaneously and in a balanced manner within a cell, preserving homeostasis [118]. Under normal physiological conditions, mitochondrial fission is required for organelle distribution during mitotic cell division [119]. In post-mitotic neurons, fission promotes transport and distribution to distal neurites [120]. Also, mitochondrial fission is used to eliminate impaired or dysfunctional mitochondria during mitophagy, the autophagic process by which defective mitochondria are selectively degraded [121,122,123] (Figure 3).

Mitochondrial dynamics are finely tuned by conserved fusion and fission proteins, whose actions are GTP-dependent. The central players are GTPases, members of the dynamin-related protein (DRP) superfamily that promote fusion (Mitofusin 1 and 2, Optic atrophy protein 1, MFN1, MFN2 and OPA1) or fission (DRP1). Here, cytosolic DRP1 is recruited by adaptors present on the outer mitochondrial membrane (OMM) called mitochondrial fission factor (MFF), human mitochondrial dynamics proteins 49 and 51 (MID49, MID51), and mitochondrial fission 1 protein (FIS1). Recruited DRP1 forms a multimeric ring complex around mitochondria, initiating separation of the inner and outer mitochondrial membranes [124,125]. Following GTP hydrolysis, the complexes constrict, resulting in mitochondrial fission and eventual division of the organelle into two [126,127,128]. This process is essentially conserved in yeast [129].

#### 3.1.2. Cyclin C Is Required for Stress-Induced and Mitochondrial Hyperfission in Mammals

**Figure 4:**
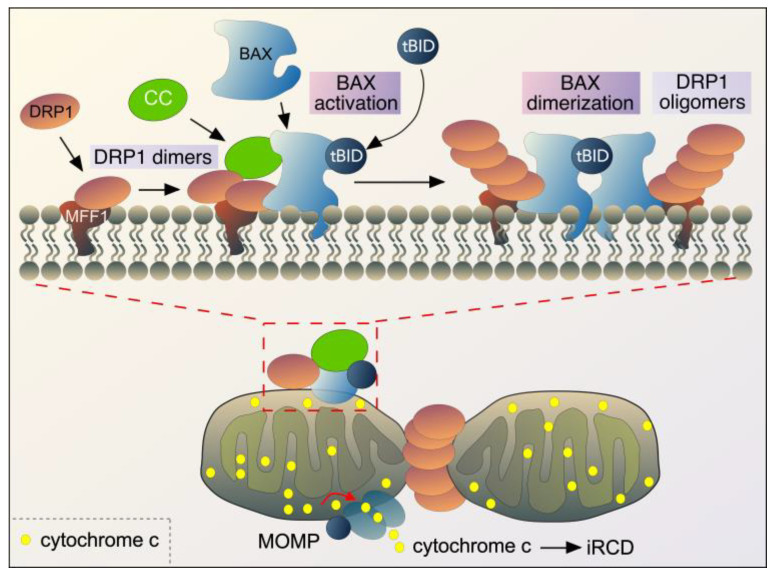
Outline of the roles of DRP1, cyclin C (CC), BAX, and tBID during intrinsic regulated cell death (iRCD) following ROS stress in mammals. The top half of the figure is an enlarged image of the box in the bottom image, depicting the interaction of key proteins at the OMM. See text for details. BAX also forms oligomers at the OMM, but this is not shown for clarity. MOMP-mitochondrial outer membrane permeabilization. Adapted from [144].

The differences between mitochondrial fission used to maintain homeostasis and that used for hyperfission are not well understood. We and others have demonstrated that the presence of cytoplasmic cyclin C following ROS stress drives cells into this hyper-fragmented state by interacting with DRP1 [92,93,130,131]. This is different to mitochondrial fission during G2 phase, in which cyclin B-CDK1 phosphorylation stimulates DRP1 activity [132,133]. Moreover, the activating phosphorylation of DRP1 does not occur following ROS stress [92]. In vitro studies revealed that cyclin C directly interacts with the GTPase domain of DRP1 [130]. Like many cyclins, cyclin C contains two cyclin boxes, a conserved structural motif of ~150 amino acid residues, which are organized into 5-helical regions [134]. The amino terminal cyclin box interfaces with CDK8/19, whereas the carboxy terminal box interacts with DRP1 [130]. This ROS stress-induced interaction triggers structural changes in DRP1, transforming it from oligomers with low GTPase activity to dimers capable of forming high-GTPase activity filaments [130]. In other words, the cyclin C–DRP1 interaction facilitates a confirmational switch that allows DRP1 to more readily bind GTP (Figure 4). Importantly, cyclin C–DRP1 interaction is both necessary and sufficient to induce mitochondrial hyperfission, as *Escherichia coli* purified GST-cyclin C introduced into permeabilized mouse embryonic fibroblasts induces mitochondrial fission in the absence of stress [92].

#### 3.1.3. Cyclin C Is Required for Stress-Induced Mitochondrial Hyperfission in Yeast

Exactly how yeast cyclin C triggers mitochondrial hyperfission complex following ROS stress is less clear. Cytoplasmic cyclin C interacts with Mitochondrial division protein 1 (Mdv1) [101], which, alongside a second adaptor (CCR4-associated factor 4, Caf4), recruits the yeast orthologue of DRP1, Dynamin 1(Dnm1), to sites of mitochondrial fission by interacting with Fis1, a conserved OMM protein [135,136]. This has led to the model in which cyclin C most likely acts early in the stress response to promote the formation of productive Fis1-Mdv1-Dnm1 complexes, leading to fission [101]. Other observations strongly support this model: firstly, artificially placing cyclin C at the mitochondria via fusion of the OMM-associating domain of Fis1 to cyclin C’s C-terminus is sufficient to induce fission even in unstressed cells [109]. Secondly, cyclin C-mediated hyperfission is not dependent upon its transcriptional duties [91,101]. Lastly, retaining cyclin C in the nucleus following ROS stress significantly reduces hyperfission.

### 3.2. Cytoplasmic Cyclin C Promotes Regulated Cell Death

#### 3.2.1. Mitochondrial Hyperfission and Intrinsic Regulated Cell Death (iRCD)

Mitochondrial hyperfission occurs in response to various acute stressors including ROS (Figure 3). This is coupled with decreased ATP production, decreased oxygen consumption, and increased mitochondrial ROS production. The outcome is decreased mitochondrial membrane potential, disrupted mitophagy, resulting in accumulation of damaged mitochondria and increased susceptibility to intrinsic regulated cell death cascades (iRCD) [137,138]. Whether mitochondrial hyperfission drives mitochondrial outer membrane permeabilization (MOMP) and iRCD remains unclear [139]. In support of this model, the absence of cyclin C, or in cis and trans mutants that keep cyclin C in the nucleus, stress-induced hyperfission and cell death is significantly reduced in both yeast and mammals alike [101]. Likewise, DRP1 GTPase activity with a dominant negative protein (DRP1^K38A^) prevents mitochondrial fragmentation and delays iRCD [140,141]. Moreover, BAX, a pro-apoptotic BCL-2 family member, which creates mitochondrial pores, moves to fission sites [142]. Together with the observation that cyclin C interacts with BAX and fission sites in mammalian cells [107], this suggests that mitochondrial hyperfission is a required step in iRCD. However, other studies have disputed this model, as cells deficient in DRP1 also undergo cell death, albeit with altered kinetics, suggesting that mitochondrial fission is not crucial for MOMP and iRCD [143]. However, more recent studies show that BAX and DRP1 directly interact, and their coupling enhances the membrane activity of both proteins [144]. Together these studies all agree that mitochondrial hyperfission occurs following ROS stress and is associated with early cellular changes observed in iRCD pathways.

#### 3.2.2. Cyclin C and Cell Death in Mammals

BAX and other members of the BCL-2 family are important regulators of iRCD in mammalian cells [145]. In healthy cells, BAX is inactive and constantly retro-translocates between cytosol and mitochondria [146]. Following ROS stress, BAX interacts with BH3 domain proteins (like tBID and BIM), which are recruited to the OMM, where they trigger a confirmational change, promoting BAX dimerization and extensive interaction with the OMM (Figure 4). Activated BAX further assembles into multiple oligomeric species, which form supramolecular structures within growing membrane pores at the MOM [147]. This results in MOMP, ultimately releasing of cytochrome c and other factors into the cytoplasm [148,149,150].

So, how do cyclin C and DRP1 fit into this model? Cytoplasmic cyclin C is required for both the activation and mitochondrial localization of BAX in mammalian cells [107]. It directly interacts with active BAX, and this interaction is dependent on the presence of DRP1 [107]. Cyclin C also interacts with DRP1 dimers, although the domains on DRP1 which interact with the second cyclin box in cyclin C remain unknown. More recently, BAX and DRP1 have been shown to directly interact [144], and future studies are needed to determine how DRP1 interacts with both cyclin C and BAX. Together, these data suggest a model where cyclin C, DRP1, BAX, and tBID form a complex essential for apoptosis. (Figure 4). Artificially stimulating cyclin C nuclear release in the absence of stress still leads to BAX–mitochondrial association, however, but does not trigger BAX oligomerization, which most likely requires tBID interaction [107]. Therefore, release of cyclin C from the nucleus is insufficient to promote cell death pathways, making cyclin C necessary but not sufficient for iRCD.

#### 3.2.3. Cyclin C and Cell Death in Yeast

*S. cerevisiae* does not encode for any known homologs of the BCL-2 family. However, expression of murine BAX in yeast induces cell death, and its cytotoxicity is dependent on its homodimerization and mitochondrial localization [149,151]. Despite this, molecular details of how yeast cells die under these circumstances remain elusive [152]. Similar to mammals, the F0F1-ATPase proton pump is essential for BAX-induced cell death in yeast [153,154]. Likewise, BAX toxicity can be rescued by co-expressing antiapoptotic members of the BCL-2 family, such as BCL-2 or BCL-xL [155]. Although BAX expression in yeast triggers cytochrome c release from mitochondria, this event is not required for cell death [156,157]. This is likely because yeast does not possess the signaling cascade of initiator and executioner caspases, which execute iRCD in mammals [158]. Moreover, the sole caspase-like protein, Yca1, is not essential for BAX-mediated cell death in yeast [159,160,161,162]. Two proteins, Uth1 and Rgl1, which are involved in cell wall strengthening and iron metabolism, respectively, do promote BAX-induced cell death, but their mechanism of action remains unknown [163,164,165].

Numerous key features of iRCD observed in mammalian cells are also conserved in yeast [166]. Interestingly, cells lacking cyclin C display remarkable resistance to stress-induced cell death, a phenomenon observed in both yeast and mammalian systems [92,167]. Although the precise role of cyclin C in this context is not fully understood, simply enhancing cyclin C-mediated mitochondrial fission through overexpression alone is insufficient to trigger RCD [101]. This suggests that an additional signal(s) is necessary to initiate RCD, which might come from another protein localized to the mitochondria during the stress response or through a stress response pathway that modifies cyclin C post-translationally. This important distinction sets cyclin C apart from other known inducers of mitochondrial RCD. For example, ectopic targeting of p53 or the BH3 family member Bax to the mitochondria can effectively induce cell death in non-stressed mammalian cells. In contrast, there is conflicting evidence for the role of Ybh3, the sole BH3 protein in yeast, in inducing regulated cell death [168,169,170,171]. In conclusion, while cyclin C plays an integral role in RCD in yeast, the exact mechanisms underlying its function are still not fully elucidated.

## 4. Roles of Med13 in the Cytoplasm

### 4.1. Role of Med13 in P-Body Assembly Following Starvation in Yeast

**Figure 5:**
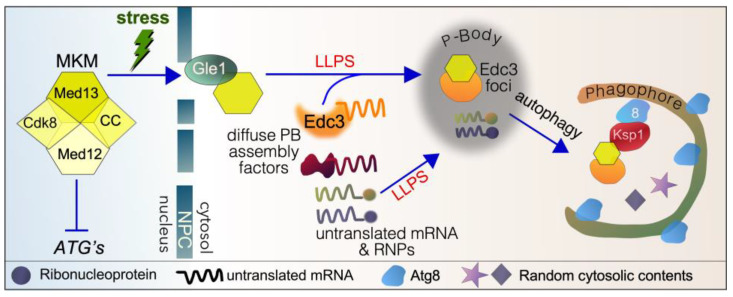
Cartoon outlining the proteins used to transport Med13 to P-bodies and then to phagophores by cargo hitchhiking autophagy in yeast. See text for details. *ATG*’s—autophagy-related genes, LLPS—liquid–liquid phase separation, 8—Atg8, NPC—nuclear pore complex, MKM—Mediator kinase model.

In the yeast model system, Med13 translocates to the cytoplasm following TOR1 inhibition triggered by either nitrogen starvation or treatment with rapamycin [44,111]. Here, it is required for processing body (P-body) assembly and the autophagic degradation of a subset of P-body assembly factors (Figure 5) [112]. P-body assembly is a conserved mechanism used to store and protect mRNA during stress (reviewed in [172,173,174]). However, the biological significance of P-body formation and stress survival is unclear and a key area of interest in this research field. In yeast, mutants defective in P-body assembly factors are less able to survive extended periods of quiescence [175], suggesting that their formation is a survival response. Likewise, *med13∆* cells are less able to survive extended periods of nitrogen starvation [44]. It remains unknown if mammalian MED13 or MED13L are required for P-body formation. Given the conservation of cyclin C’s secondary job, it is highly likely that mammalian MED13 will follow suit.

#### 4.1.1. P-Body Assembly Following Stress

Inhibiting mRNA translation is a critical strategy cells employ to manage protein levels effectively. Under stress, cells strategically downshift the production of proteins vital for growth while ramping up those necessary for adaptation. A pivotal mechanism for blocking mRNA translation is the formation of P-bodies. These highly conserved, membrane-less structures within the cytoplasm are reservoirs that contain non-translating mRNA-protein complexes (mRNPs) [176,177]. Initially, it was believed that mRNAs within P-bodies were simply stored for future translation or subjected to degradation [173,178,179,180]. However, subsequent studies have revealed that P-bodies are not crucial for mRNA degradation [181]. Instead, they are increasingly recognized as being temporary storage sites for translationally repressed mRNAs that remain ready for activation when conditions improve [182].

The assembly of P-bodies occurs through a process known as liquid–liquid phase separation (LLPS), creating dynamic droplets that are distinctly separate from the surrounding cytoplasm [183]. Their assembly benefits cells, as P-body formation occurs more rapidly than changes in the transcriptional or translational programs. Moreover, the rapid disassembly of P-bodies after the stress abates rapidly provides cells with ready-to-go translation components. The proteins that reside within P-bodies are predominantly RNA-binding proteins or factors with low sequence complexity that contain prion-like domains (PLDs). These PLDs possess the remarkable ability to undergo spontaneous conversion into aggregated states that serve as templates for the recruitment of additional proteins.

#### 4.1.2. Med13’s Role in P-Body Assembly in Yeast

In yeast, P-bodies are consistently present under normal physiological conditions, but their size and number notably increase in response to various stressors, such as nitrogen and carbon starvation [183]. The assembly of P-bodies is fundamentally dependent on several conserved decapping proteins, including Dcp1/Dcp2, Edc3, and Dhh1, along with the Pat1–Lsm1–7 complex and Xrn1 [173]. Edc3 serves as a crucial scaffold for P-body assembly, by leveraging its multivalent interactions [184,185,186,187,188]. Med13 is essential for P-body assembly following nitrogen starvation; the stark reduction in Edc3 foci in *med13Δ* cells underscores its importance [112]. The mechanistic details of why Med13 is required for P-body formation remain to be completely elucidated; however, it is clear that the PLD domain of Med13 is indispensable for this process [112]. The exact function of these polyQ/N tracts is not well understood, but it has been suggested that the domain can interact with itself or with other polyQ/N regions to promote the aggregation of mRNPs [188].

These studies indicate that Med13 plays a significant role in promoting LLPS of P-body proteins. Furthermore, Med13 contains two motifs strongly linked with LLPS: an RNA recognition motif (RRM) and a large intrinsically disordered region (IDR). IDRs are recognized as key drivers of LLPS, as they can simultaneously create multiple interactions with other components [189]. Additionally, the amino acids within IDRs are more exposed and accessible to post-translational modifications, which are critical regulators of biomolecular phase separation [190,191,192]. This suggests that Med13 itself may undergo LLPS, a direction future studies should pursue.

### 4.2. Med13’s Role in Cargo Hitchhiking Autophagy in Yeast

Cytoplasmic Med13 is a cargo of cargo hitchhiking autophagy (CHA) in yeast [44,111]. This hybrid autophagy pathway was only discovered in 2024 [19], and the only reports to date are for *S. cerevisiae.* It remains to be seen whether similar pathways exist in higher eukaryotes. Given the conserved nature of many of the players, it seems highly likely that a similar mechanism exists.

#### 4.2.1. Outline of Autophagy Pathways

Autophagy is a highly conserved core molecular pathway used to maintain homeostasis by intracellular degradation of random cargos, dysfunctional proteins, and organelles. Details of the processes involved have been discussed in many recent excellent reviews by experts in the field [5,193,194]. In short, double-membrane vesicles called autophagosomes engulf proteins and organelles. Cargo-laden autophagosomes fuse with vacuoles (in yeast and plants) or lysosomes (in metazoans), resulting in the degradation of captured contents by resident hydrolases [195,196]. From yeast to humans, ordered steps define the process of autophagy. The first stage is initiation, followed by the double-membrane phagosome’s nucleation, expansion, and elongation. Thereafter, the phagosome closes, forming an autophagosome, which fuses with lysosomes. Here, resident proteases degrade autophagosomes and their cargo, providing fuel for cells [5,197,198,199].

Mechanistically, all autophagy pathways are classified according to cargo type and the lysosomal delivery system employed, resulting in three major groups: macroautophagy, microautophagy, and chaperone-mediated autophagy (CMA). Macroautophagy and microautophagy are conserved pathways [200] with macroautophagy being further classified into selective and non-selective pathways [201,202,203]. Importantly, these pathways play crucial roles in cellular physiology and are protective against a wide variety of diseases, including neurodegeneration, cancer, infections, and cardiovascular disorders [194,204,205,206].

Given the conservation of autophagy, *S. cerevisiae* is a leading model organism for deciphering molecular details that define macroautophagy pathways. In this system, autophagic pathways fall into two subclasses: selective and non-selective (bulk) autophagy. Bulk autophagy is predominantly upregulated following Target of Rapamycin complex 1 (TORC1) inhibition, triggered by nutrient stress, and degrades superfluous random cytosolic proteins and organelles. In contrast, selective autophagy pathways maintain cellular homeostasis when TORC1 is active by degrading damaged organelles and dysfunctional proteins. Here, selective autophagy receptors mediate cargo recognition by Atg8, a conserved protein required for phagophore expansion.

#### 4.2.2. Med13 Is Degraded by Cargo Hitchhiking Autophagy in Yeast

Very recently, we and others have discovered a new hybrid autophagy mechanism, coined cargo hitchhiking autophagy (CHA) in yeast [44,111,207,208] (reviewed in [19]). This mechanism is induced during starvation and utilizes autophagy components from both bulk and selective autophagy pathways. CHA uses receptor proteins to deliver selected cargos, including a subset of ribosomal proteins, TFs, and TIFs, to phagophores. In CHA, various cargo hitchhiking receptors link their cargos to lipidated Atg8, located on growing phagophores. These phagophores are built to capture random cytosolic content during starvation (bulk autophagy), and the prevailing model is that CHA receptors hitchhike onto Atg8 in these preformed phagophores.

Med13 is one of the cargos degraded by CHA following nitrogen starvation using Ksp1 as a receptor [44,111]. The known players used to mediate Med13 from the nucleus to awaiting phagophores are outlined in Figure 5 and discussed in the sections below. More recently, we discovered that Med13 is also required for the autophagic degradation of both Edc3 and Dhh1 following nitrogen starvation. Since Med13 does not direct the autophagic degradation of Xrn1, this suggests that Med13 serves as an exclusivity factor that differentiates among various P-body components [112]. Given the conserved nature of P-body assembly factors and autophagy pathways, it will be exciting to determine whether this mechanism is conserved in higher eukaryotes.

#### 4.2.3. Snx4 Promotes CHA in Yeast

CHA was previously named Snx4-assisted autophagy, as this conserved sorting nexin is required for the degradation of all identified CHA cargos to date [19,44,207,208,209]. Sorting nexins are a family of conserved phosphoinositide-binding proteins that play fundamental roles in orchestrating cargo sorting through the endosomal network. As such, human SNX4 is implicated in a variety of synaptic processes [210,211]. Significantly, SNX4 has already been linked with the etiology of AD, with SNX4 protein levels being decreased by 70% in the brains of severe Alzheimer’s disease (AD) cases [212].

Sorting nexins are recruited to endosomal membrane domains by a phox homology (PX) domain that recognizes phosphatidylinositol-3-phosphate (PtdIns3P) [209]. Snx4 also contains two Bin–Amphiphysin–Rvs (BAR) domains that sense membrane curvature and tubulate vesicles [213]. The molecular role of Snx4 (Atg24) in autophagy is also related to membrane bending as it stabilizes and drives the opening of the inner membrane rim of non-selective phagophores in yeast [214]. The net effect is a phagophore membrane with a wide enough opening to accommodate large cargos, including ribosome and proteasome subunits [214,215,216,217].

#### 4.2.4. CHA Uses Phagophores Built by the Atg17 Scaffold Complex

In CHA, specific cargos, including Med13, are recruited to growing phagophores that are primarily used to degrade random cytosolic contents by non-selective (a.k.a. bulk) autophagy. These phagophores are triggered by starvation stress. Their formation is dependent on the Atg17 scaffold complex, which consists of the Atg17 scaffold and two regulatory proteins, Atg29 and Atg31 [218]. This process differs from selective autophagy, where phagophores are constructed using the Atg11 scaffold [219]. Selective autophagy pathways play crucial roles in maintaining homeostasis under physiological conditions. They are responsible for removing dysfunctional proteins (such as in mitophagy, ER-phagy, and ribophagy), protein aggregates (aggrephagy), and pathogens like viruses and bacteria (xenophagy) in higher eukaryotes [220]. It is likely that Med13-Edc3 is not the only cargo captured in Atg17-built phagophores. Instead, these phagophores probably contain random cytosolic contents alongside other cargo that may hitch a ride, including a subset of ribosomal proteins [208].

The formation of the phagophore assembly site (PAS) for both selective and non-selective autophagy depends on LLPS [221,222]. During starvation, the Atg1 complex comprising Atg1, Atg13, and a trimeric Atg17 scaffold undergoes LLPS [223]. This fluidity is vital for recruiting downstream components necessary for autophagosome formation. As mentioned above, P-body assembly occurs via LLPS. How these two biomolecular condensates (BMC) interact remains unknown. One intriguing possibility is that Med13 could play a role, possibly acting as a conduit between these two BMCs.

#### 4.2.5. Ksp1 Is the Selective Autophagy Receptor Proteins for Med13 in CHA

Although CHA uses phagophores built for the degradation of random cytoplasmic contents, cargo recognition is dependent upon an autophagic receptor protein [19]. The autophagic receptor for the selective degradation of Med13 is Ksp1. Typical of autophagic receptors, Ksp1 interacts with the phagophore-bound protein Atg8 via its conserved Atg8 interaction motif (AIM) [111,224]. Ksp1 is a casein-like kinase that negatively regulates autophagy in replete media [225,226,227,228,229]. However, its role as a receptor protein is kinase-independent, illustrating its dual and opposing roles in autophagy. These studies support the emerging concept that proteins can perform two distinct functions, referred to as “day and night jobs” [230,231], which various external or intrinsic stimuli can trigger. Ksp1 also associates with ribosomal and PB proteins [232,233], though the function of Ksp1 here remains unknown. Interestingly, Ksp1 does not directly interact with Edc3. Instead, Med13 performs this role, suggesting that it may act as a conduit between Edc3 and Ksp1 [111].

#### 4.2.6. The Nucleoporin Gle1 Is Required for CHA of Med13

Cyclin C is a small 33 kDa protein that can diffuse through the nuclear pore complex (NPC) without the assistance of active transport by exportins. In contrast, Med13 is a larger scaffold protein, approximately 150 kDa in size, which requires active transport to cross the nuclear–cytoplasmic barrier. However, our studies have shown that β karyopherin exportins are not needed for Med13’s transit through the NPC. Similarly, Dpb5 and the Mex67-Mtr2 heterodimer, which can transport cargo across the NPC, do not play a role in this process. Instead, Gle1, a conserved component of cytoplasmic NPC fibrils, is essential for Med13’s passage across the NPC [44].

GLE1 is a mobile nucleoporin in mammalian cells [234], suggesting that it could play a similar role in the nuclear export of Med13. Interestingly, in yeast Med13 remains in the nucleus following nitrogen starvation in *snx4∆* mutants [44]. This observation implies that Snx4 may communicate with the NPC, potentially by interacting with Gle1 during the transfer of Med13 as it exits the NPC. Future experiments will be necessary to investigate this idea further.

## 5. Diseases Associated with the MKM

### 5.1. Tumor-Suppressive Roles of Cyclin C–CDK8/19

#### 5.1.1. Notch Signaling

The cyclin C locus (*CCNC*) maps to 6q16.2 [235]. Deletions of 6q16.2 are frequently found in blood cancers and solid tumors, hinting at a tumor suppressor role for cyclin C [236,237,238,239]. Mechanistic details have started to emerge on how cyclin C acts as a tumor suppressor in T-cell acute lymphoblastic leukemia (T-ALL) [240] Here, aberrant *NOTCH1* expression is a causative factor in T-ALL development [241]. In the canonical NOTCH signaling pathway, ligand-activated Notch1 receptors are cleaved, releasing a Notch intracellular domain (ICN1) which migrates to the nucleus. Here, ICN1 initiates transcription of downstream target genes by interacting with the DNA-bound CSL–co-repressor complex (CBF-1, Suppressor of Hairless, and LAG-1). By displacement of co-repressors and recruitment of the transcriptional co-activator protein Mastermind-like (MAML), this transforms CSL into a co-activator complex, resulting in transcription of Notch target genes [242,243].

Post-translational modifications are well known to regulate Notch activity [244]. These include ubiquitination and UPS-mediated destruction of the ICN1. Although the exact role of ICN1 destruction is not clear, it has been suggested that the Notch degradation complex is recruited to activated promoters and may function to disassemble active enhancer complexes [67]. Importantly, the cyclin C–CDK8 kinase phosphorylates ICN1, marking it for degradation. In the hematopoietic lineage, the major rate-limiting function of cyclin C is to suppress ICN1. Loss or heterozygosity of cyclin C disrupts this regulatory mechanism, enhancing Notch1 activity, which promotes T-ALL progression [240].

Notch1 activation is present in many cancers (acute lymphoblastic leukemia, non-Hodgkin’s lymphoma, and prostate cancer, amongst others). As 6q16.2 deletion is associated with these cancers, this suggests that the cyclin C–CDK8-mediated degradation of ICN1 likely plays a role [239,245,246,247,248,249]. It should also be noted that in some cellular contexts, Notch1 functions as a tumor suppressor [250]. The role of cyclin C in these contexts, especially with regard to ICN1 degradation, remains unknown [240,251,252]. This is important to understand, given the use of CDK8/19 inhibitors that are currently in clinical trials (see below).

#### 5.1.2. JAK-STAT Signaling

Cyclin C also plays a tumor suppressor role in anaplastic thyroid tumors [253]. This is maybe not surprising as 6q16.2 is lost in 33% of poorly differentiated thyroid tumors and 27% of anaplastic malignancies [254]. In mouse models of thyroid neoplasia [255], deletion of cyclin C alone in the thyroid only stimulates a modest increase in hyperplastic growth. However, in combination with ablation of the *PTEN* tumor suppressor [256], the thyroid size increases dramatically, eventually killing the animal [253]. Despite the increase in ROS production throughout thyroid cancer development, it is not the mitochondrial role of cyclin C that is involved. Instead, alterations in cyclin C–CDK8-mediated phosphorylation of STAT-3 result in misregulated JAK-STAT signaling, which in turn destabilizes the tumor suppressors p53 and p21 [107,257].

### 5.2. Oncogene Roles of Cyclin C–CDK8/19

CDK8 was first reported as a putative oncogene in colorectal cancer in 2012 [258]. In this context, CDK8 mediates the aberrant activation of the Wnt/β–catenin signaling pathway. Additionally, the phosphorylation of various transcription factors, such as Smads, STAT1, and NFκB, by CDK8 is frequently dysregulated in many other types of cancer [53,77,259]. This suggests that cancers associated with CDK8 are predominantly driven by transcriptional dysregulation [260]. Currently, mutations in both CDK8 and CDK19 have been linked to over 100 malignancies, including cancers of the colon [258,260], breast [261], prostate [262], pancreas [263], skin [264], and blood [86]. It comes as no surprise then that modulating the transcriptional activity of the MKM has emerged as an attractive therapeutic strategy for treating cancer. Consequently, specific inhibitors targeting CDK8/CDK19 are currently being tested in various clinical trials [259].

Cyclin C–CDK8/19 is not alone in having dual roles with seemingly contradictory functions. Extensive research has determined that other tumor suppressors, including Rb, PTEN, and FOXO, also act as double agents with oncogenic roles [265]. These roles can be dependent upon cell type and/or post-translational modification events. A classic example in this case is Notch (described above), which has an oncogenic role in T-ALL and a tumor suppressor role in squamous epithelial cells [266,267]. Interestingly, a recent survey identified 73 unique tumor suppressors with oncogenic potential. Like cyclin C–CDK8/19, many of these double agents are transcription factors, whose oncogenic potential is realized by interaction with different proteins [268]. It is therefore critical to determine the context-dependent role of these double agents before treatment.

### 5.3. Role of Cyclin C in TDP-43-Mediated Cell Death

#### 5.3.1. Role of TDP-43 in Maintaining Homeostasis

Transactive Response Binding Protein 43 (TARDBP, hereafter referred to as TDP-43) is a ubiquitously expressed protein well conserved among mammals and vertebrates [269]. It contains two RNA-recognition motifs (RRMs) that can bind UG/TG-rich single-stranded or double-stranded DNA/RNA. As such, TDP-43 performs various functions in transcriptional repression, pre-mRNA, and alternative splicing [270,271]. The C-terminus is essential for solubility and regulates protein–protein interactions. It also contains a prion-like domain where many disease-associated mutations are located [272]. TDP-43 also contains a nuclear localization sequence and as such is predominantly located in the nucleus. However, in non-pathogenic states it also shuttles to the cytoplasm to carry out additional functions, including mRNA stability and transport, translation, the stress response, and autophagy regulation (see [273] for a recent review). Here, it interacts with many subcellular compartments, including the endoplasmic reticulum and stress granules. Also, a small amount of TDP-43 is transported into mitochondria via the TOM20 and TIM22 complex [274] to stabilize mitochondrial transcript intermediates [275]. Therefore, the normal physiological functions of TDP-43 are particularly important for cell survival. Importantly, under normal physiological conditions, the low cytoplasmic levels of TDP-43 are finely tuned by a negative-feedback mechanism [276]. In the mitochondria, this includes TDP-43 degradation by mitochondrial proteases [277]. There is still much debate in the field on whether TDP-43 is internalized into the mitochondria, but it is clear that mitochondria play a role in response to aberrant TDP-43 [278].

#### 5.3.2. Aberrant Cytoplasmic Roles of TDP-43

**Figure 6:**
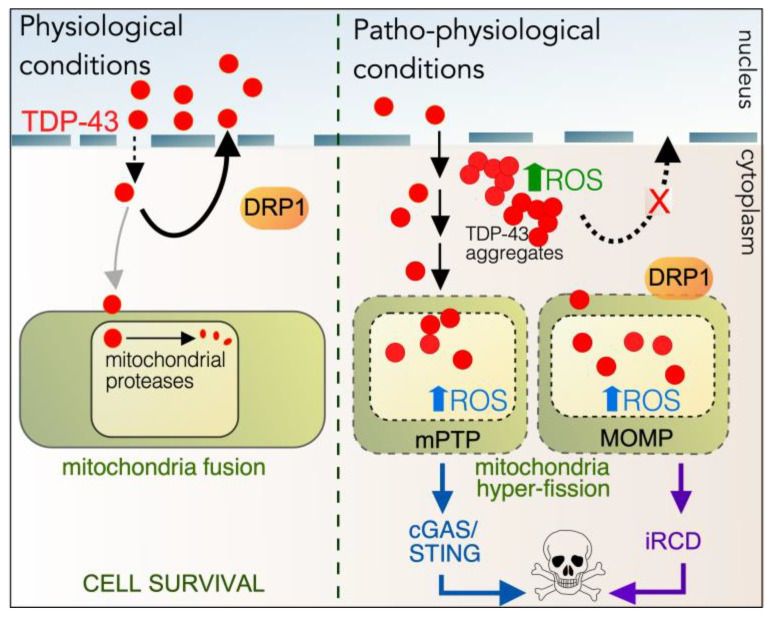
Proposed roles of TDP-43 in physiological and patho-physiological conditions. Left hand panel: In unstressed cells, TDP-43 is predominantly a nuclear protein, but it also shuttles to the cytoplasm to play various roles, including translation. Most TDP-43 is shunted back into the nucleus mediated by its nuclear localization signal. Some TDP-43 also enters the matrix of mitochondria, where it regulates mitochondrial translation before being destroyed by resident proteases. DRP1 does not co-localize at the OMM, and mitochondrial dynamics are normal, with most cells exhibiting a fused morphology. Right-hand panel: Mutant TDP-43 is not well retained in the nucleus, resulting in cytoplasmic aggregates, increasing intracellular ROS. More TDP-43 is also found in mitochondria, which also trigger increase in ROS, resulting in cell death via the cGAS-STING and/or iRCD pathways. DRP1 co-localizes to the OMM inducing hyperfission and MOMP.

A hallmark of ALS and other proteinopathies is the aberrant localization of TDP-43 into cytoplasmic aggregates (Figure 6, [279,280]). The diseased form of TDP-43 is predominantly found in the cytoplasm, where it is hyperphosphorylated, ubiquitinated, and proteolytically cleaved into C-terminal fragments (CTFs) that are devoid of a functional nuclear localization signal [281,282,283]. Cytoplasmic accumulation of CFTs results in nuclear depletion and aggregate formation sequestering full-length TDP-43 and increasing cellular reactive oxygen species (ROS) [284,285]. Aggregate accumulation in TDP-43 toxicity has been associated with autophagy impairment [286,287,288,289,290].

The mitochondrial accumulation of TDP-43 is also observed in TDP-43 toxicity. This results in increased DRP1-mediated mitochondrial fragmentation. Consistent with this are several observations: inhibiting TDP-43 localization to mitochondria or preventing fission blocks ALS-associated pathologies [277,291,292,293,294]; decreased MFF1 and 2 and increased DRP1 expression occur in motor neurons in a mouse model of ALS [295]; mitochondrial hyperfission has also been observed in the post-mortem neurons of patients diagnosed with ALS and other proteinopathies, as well as in cell cultures [284,285,291,292,294].

The accumulation of mitochondrial TDP-43 also triggers cell death by pathways that converge at the mitochondria (Figure 6, [275,292]). TDP-43 mitochondrial overload induces mitochondrial outer membrane permeabilization (MOMP), allowing iRCD). Alternatively, the mitochondrial permeability transition pore (mPTP) opens releasing mitochondrial DNA (mtDNA) and mtRNA:DNA into the cytoplasm. This stimulates the cyclic GMP-AMP synthase (cGAS)/Stimulator of Interferon Genes (STING) cell death pathway [294]. Therefore, understanding the molecular details underlying the mitochondrial invasion by TDP-43 represents an important step in devising therapeutic treatments for TDP-43 proteinopathies.

#### 5.3.3. Cyclin C and TDP-43

Using the established yeast model of TDP-43 pathology, it has been shown that cyclin C, but not Med13, promotes TDP-43-mediated cell death [296]. Likewise, the deletion of Dnm1 rescues TDP-43 toxicity, pointing to the potential mitochondrial fission role of cyclin C in TDP-43 toxicity. Furthermore, cyclin C translocates to the cytoplasm following TDP-43 overexpression. The free radical scavenger, N-acetyl-cysteine (NAC), inhibits cyclin C cytoplasmic relocalization. These studies suggest the possibility that cytoplasmic cyclin C may promote mitochondrial hyperfission and cell death upon expression of mutant TDP-43. This is exciting as cyclin C is a new player in TDP-43 biology and could represent a new target for drug therapies. This is important as currently there are no effective cures for ALS, and patient survival upon diagnosis is around 3–5 years [297].

### 5.4. Diseases Associated with MED13 Biology

In mammals, the MKM contains paralogues of its members, except cyclin C, namely MED12L, MED13L, and CDK19 [27]. The biological roles of these paralogues remain poorly understood, but they appear to be functionally distinct [298]. Many mutations in MED13L result in MED13L syndrome, a disease characterized by a range of symptoms that vary in severity. These include intellectual disability, facial dysmorphism, hypotonia, congenital heart disease, and speech and motor delays [299,300,301,302]. In MED13L, mutant fibroblast cyclin C is aberrantly released into the cytoplasm, leading to mitochondrial fragmentation and increased mitochondrial dysfunction [303]. Variants in CDK8, MED13, MED12*,* and MED12L are also associated with neurodevelopmental disorders [73,304,305,306,307,308], with cyclin C’s role here being unknown. In addition, mutations in MED12 are linked with uterine leiomyomas [309]. Interestingly, all the MKM neurodevelopmental disorders are phenotypically similar, indicating a probable overlap in pathogenic mechanisms.

## 6. Conclusions

The MKM has evolved in the last 20 years from being a transcriptional regulator to mediating mitochondrial hyperfission, regulated cell death, P-body assembly, and cargo hitchhiking autophagy. These stress-dependent secondary roles trigger the nuclear release of either cyclin C or Med13, following ROS or starvation stress, respectively. This enables cells to quickly respond to unfavorable environmental cues, facilitating communication and regulatory functions between cellular compartments.

The ability of transcriptional regulators to shuttle between the nucleus and cytoplasm in response to cellular cues is well established. For example, upon activation the transcription factor NF-κB moves into the nucleus, where it triggers transcription of genes involved in survival, inflammation, and the immune response [310]. More unusual though are proteins like cyclin C and Med13 that play different roles in different compartments. A great example is the nuclear transcription factor TAF7 that chaperones its target RNAs from the nucleus to polysomes in the cytoplasm, where it contributes to the regulation of translation [311]. Another dual-role transcription factor is p53, which also regulates translation primarily through its interaction with components of the translation machinery [312]. p53 also migrates to the mitochondria, mediating iRCD primarily through direct protein–protein interactions with BCL-2 family proteins [313]. TDP-43, as discussed above, plays roles in splicing in the nucleus and translation in the cytoplasm. Likewise, alpha-synuclein, the protein long associated with Parkinson’s disease and Lewy body dementia, activates a calcium pump in cell membranes [314] and modulates P-bodies in the cytoplasm [315]. These examples suggest that other proteins may have dual roles that are currently unknown. It is important to investigate, not only from the point of view of unravelling complex molecular pathways but also for targeted drug design, which has been dominated by the “one target, one drug” concept.

The conservation of the roles of cytoplasmic cyclin C are remarkable, with minor differences being observed between yeast and mammalian cells. Maybe the largest difference is the observation that in yeast most of cyclin C shuttles to the cytoplasm [91,101], whereas in mammalian cells only 10% of cyclin C is observed at this new subcellular address after stress [92]. The most logical explanation for these differences is that in mammals, cyclin C positively and negatively regulates an equal number of SRGs, whereas in yeast it is mainly a negative regulator [316].

## 7. Future Prospectives

The most important gaps in our knowledge of MKM biology following stress are related to the determination of whether the cytoplasmic role of Med13 found in yeast is conserved. Yeast Med13 and MED13/13L in mammals share structural features, including large IRDs and prion-like domains. As these motifs are characteristic of proteins able to undergo LLPS, this suggests a strong possibility that Med13 may also contribute to BMC formation after stress. Consistent with this, the PrLD of yeast Med13 is required for P-body formation [112]. Future experiments need to address whether this cytoplasmic role of Med13 is conserved. If MED13 and MED13L do relocate to the cytoplasm in mammals, then it would be important to ask whether they also undergo autophagy. This avenue of research could potentially lead to the discovery of CHA in mammals. As autophagy is a key regulator of homeostasis, including the removal of aggregate proteins, [317] this line of research could provide valuable insights into the molecular basis of proteinopathies.

Lastly, in addition to guiding studies in higher eukaryotes, understanding the stress response in yeast is important in its own right. In the US, the wine industry generates billions of dollars of revenue annually, and craft beer brewing alone has recently emerged as a multibillion-dollar industry [318,319]. *S. cerevisiae* encounters different stresses during brewing, such as hyperosmotic, ethanol, and thermal stresses [320,321]. In addition, in fed-batch-operated industrial bioreactors, pockets of yeast can face glucose starvation, resulting in negative consequences for production performance [322]. Understanding and manipulating the yeast stress response could spur industrial advancements and increase fermentation yield efficiency [323,324,325]. Ultimately, the MKM, particularly cyclin C and Med13, plays a critical role in a variety of diseases, emphasizing the need for further investigations of both the mechanistical aspects and the therapeutic potential.

## Data Availability

No new data were created or analyzed in this study.

## Abbreviations

The following abbreviations are used in this manuscript:

AMPK	Adenosine Monophosphate-activated Protein Kinase
ALS	Amyloid Lateral Sclerosis
AIM	Atg8 Interacting Motif
BAR	Bin–Amphiphysin–Rvs
BMC	Biomolecular Condensate
cGAS	Second messenger cyclic GMP–AMP
ChIP	Chromatin Immunoprecipitation
CWI	Cell Wall Integrity pathway
cMED	Core Mediator Complex
HAD	Holoenzyme-associating Domain
IMM	Inner Mitochondrial Membrane
IDR	Intrinsically Disordered Region
iRCD	Intrinsic Regulated Cell Death
LLPS	Liquid–liquid Phase Separation
MKM	Mediator Kinase Module
OMM	Outer Mitochondrial Membrane
MOMP	Mitochondrial Outer Membrane permeabilization
MEFs	Mouse Embryonic Fibroblasts
NAC	N-acetyl-cysteine
mPTP	Mitochondrial Permeability Transition Pore
mRNPs	Non-translating mRNA-protein complexes
NPC	Nuclear Pore Complex
PI(3)P	Phosphatidylinositol-3-phosphate
PX	Phox Homology
PrLD	Prion-Like Domains
P-Body	Processing Body
RCD	Regulated Cell Death
RNPs	Ribonucleoproteins
RNAPII	RNA Polymerase II
RRM	RNA Recognition Motif
STING	Cyclic GMP–AMP receptor stimulator of interferon genes
SRGs	Stress Response Genes
T-ALL	T-Cell Acute Lymphoblastic Leukemia
TFs	Transcription Factors
TIFs	Translation Initiation Factors
UPS	Ubiquitin–Proteasome System
